# Risk of diabetes and expected years in life without diabetes among adults from an urban community in India: findings from a retrospective cohort

**DOI:** 10.1186/s12889-024-18465-2

**Published:** 2024-04-15

**Authors:** Palak Sharma, T.R. Dilip, Anjali Kulkarni, Udaya Shankar Mishra, Yogesh Shejul

**Affiliations:** 1 Department of Family and Generations, International Institute for Population Sciences, Mumbai, 400088 India; 2 Medical Division, Bhabha Atomic Research Center, Mumbai, 400088 India; 3 Department of Bio-statistics and Epidemiology, International Institute for Population Sciences, Mumbai, 400088 India

**Keywords:** Cohort study, Type 2 diabetes, Disease progression, Electronic medical records (EMR), Life table

## Abstract

**Background:**

Diabetes prevalence has increased over the past few decades, and the shift of the burden of diabetes from the older population to the younger population has increased the exposure of longer durations in a morbid state. The study aimed at ascertaining the likelihood of progression to diabetes and to estimate the onset of diabetes within the urban community of Mumbai.

**Methods:**

This study utilized an observational retrospective non-diabetic cohort comprising 1629 individuals enrolled in a health security scheme. Ten years of data were extracted from electronic medical records, and the life table approach was employed to assess the probability of advancing to diabetes and estimate the expected number of years lived without a diabetes diagnosis.

**Results:**

The study revealed a 42% overall probability of diabetes progression, with age and gender variations. Males (44%) show higher probabilities than females (40%) of developing diabetes. Diabetes likelihood rises with age, peaking in males aged 55–59 and females aged 65–69. Males aged 30–34 exhibit a faster progression (10.6 years to diagnosis) compared to females (12.3 years).

**Conclusion:**

The study’s outcomes have significant implications for the importance of early diabetes detection. Progression patterns suggest that younger cohorts exhibit a comparatively slower rate of progression compared to older cohorts.

## Highlights

**High Diabetes Progression Rates**: A striking finding is that approximately 2 out of every 5 individuals aged 30 years and older advanced to diabetes within a mere decade.

Another intriguing observation is the gender disparity in diabetes progression. Males, in particular, exhibited a notably higher likelihood of transitioning to diabetes during this timeframe.

When we delve into age-specific data, it was found that among women aged 30–34 years, there’s an average of 12.3 years expected to pass before a diabetes diagnosis is made. However, for their male counterparts, this waiting time shrinks significantly to just 10.6 years. This discrepancy indicates a faster progression toward diabetes among males.

## Background

The non-communicable diseases (NCD) are the leading cause of premature morbidity and mortality. Globally, there are around 537 million adults aged 20–79 years currently living with diabetes and India alone accommodates around 77 million diabetic people [1]. The prevalence of diabetes has increased many fold in recent decades worldwide, yet among major NCDs, it has usually been underestimated in terms of the cause of death. Diabetes is considered to have little impact on mortality directly when compared to morbidity as it mainly expedites the risk of microvascular complications [2] and various other severe chronic conditions [3]. Only a minority of patients uniquely die due to diabetes [3, 4].

The evolving demographics, epidemiology, and longer lifespans in recent decades have raised concerns about the quality of life for individuals with diabetes. Hence, understanding disease progression and a life-course perspective is vital for designing studies that evaluate intervention impacts in terms of preventing and delaying diabetes [5, 6].

Observing diabetes progression across different age groups and genders reveals the significant impact of demographic variables on both the development and duration of morbidity. It is known that blood glucose concentrations tend to rise with age [7], and previous national surveys reported that 54% people of those who develop diabetes were in the most productive years of their lives (< 50 years) with a higher risk of developing chronic complications of diabetes [8, 9]. Most studies, both in India and internationally, indicate a higher prevalence and risk of diabetes among men [10–19], while a few studies reported otherwise [4, 20]. The shift in diabetes onset among the young and increased life expectancy for those with diabetes has prolonged the duration of time spent with diabetes and related sufferings [21, 22].

Determining the onset of diseases, particularly for asymptomatic conditions like diabetes with delayed symptom manifestation, poses a significant challenge. Hence, some studies diverge by reporting the age at diabetes detection rather than onset, introducing variations [22]. Also, numerous studies have employed the life table approach to explore the transition from non-diabetic states to diabetes, assessing the proportion of remaining life with and without diabetes [21, 23, 24]. However, these studies couldn’t estimate diabetes onset and progression patterns in real-time cohorts with continuous follow-up and clinical diagnosis using laboratory tests, as many longitudinal studies in India lack consistent follow-up and were primarily conducted cross-sectionally with an interval of eight to ten years [12, 25–27]. With current technological advancements, early detection and screening for diabetes is crucial for preventing or delaying its onset [28]. Hence, this study’s main objective is to assess the probability of diabetes progression in a diabetes-free cohort and estimate the remaining diabetes-free life years in the population.

## Methods

This retrospective cohort study examines diabetes progression among beneficiaries of a Contributory Health Service Scheme (CHSS), using Electronic Medical Records (EMRs) from a Government hospital in urban Mumbai. The beneficiary population features uniform and universal access to healthcare under CHSS, with moderate to high educational attainment and economic stability.

In 2010-12, about 30,463 CHSS beneficiaries were registered with the hospital, and clinical tests revealed thatof these, 835 beneficiaries aged 30 years and above were newly diagnosed with diabetes. Hence, a sample double the size of these cases i.e. 1669 beneficiaries, matched for age and sex, without diabetes diagnosis during this period, was followed up for next ten years (January 2012 to December 2021) to investigate diabetes progression and its patterns. Detailed population characteristics are available elsewhere [29]. Further, 40 beneficiaries were lost to follow-up in the first year (2012) and were excluded from the analysis due to the unavailability of data on the last visited date. Hence, the analysis in this study is restricted to a total of 1629 beneficiaries.

The main clinical endpoint was the transition from non-diabetic status to clinically diagnosed diabetes. The study adopted the American Diabetes Association 2021 definition to categorize individuals with diabetes [30]. An individual is considered to have diabetes if the fasting plasma glucose (FPG) ≥ 126 mg/dl (7.0 mmol/l), or postprandial glucose ≥ 200 mg/dl (11.1 mmol/l), or HbA1c ≥ 6.5% or that individual is on anti-diabetes medication. Table 1 presents the parameters and the cut off values used for the diagnosis of diabetes in a tabular format (Table 1).

**Table 1:** Definition of diabetes and pre-diabetes as per the American Diabetes Association, 2021 [30]

Category	Fasting plasma glucose (FPG)	Postprandial glucose (PPG)	HbA1c
Normoglycemia	FPG < 100 mg/ dL	PPPG < 140 mg/ dL	HbA1c < 5.7%
Pre-diabetes	100 < = FPG < 126 mg/ dL	140 < = PPPG < 200 mg/ dL	5.7 < = HbA1c < 6.5%
Diabetes	FPG > = 126 mg/ dL	PPPG > = 200 mg/ dL	HbA1c > = 6.5%

A single decrement life table was constructed to analyze the progression of diabetes within the cohort. In this table, individuals are considered as having exited the study when they are diagnosed with diabetes or when they are lost to follow-up. Loss to follow-up, although an event that leads to exit from the cohort, is not considered a separate decrement in this particular life table.

The probability of an individual being diagnosed with diabetes is given by the formula:

$$Probability\,of\,turning\,diabetic\,in\,ten\,years\left( {nqx} \right)\, = \,{{ndx} \over {lx}}$$

Where *ndx* is the number of beneficiaries in the age group x to x + n who turned diabetic in ten years and *lx* is the number of person years contributed over the period by all individuals at risk in age group x to x + n.

Further, using the age and sex-specific probabilities of progressing to diabetes determined over the ten-year follow-up period, the study estimated the average number of years an individual is expected to live before being diagnosed with diabetes based on the life table columns and formulas. The mathematical formulas used to estimate each column of the life table have been published elsewhere [31]. This analysis provides insights into the expected age of onset of diabetes in the synthetic cohort of beneficiaries with normoglycemia across various age groups for both males and females.

## Results

### Sample characteristics

Table 2 presents the age-sex distribution of the selected synthetic cohort of beneficiaries who were followed up for a period of ten years. Of the total sample of 1629, 56% were female, and the mean age of the beneficiaries included in the study was 55.2 years. Additionally, more than one-third of the sample (36%) was aged 60 years and above, implying the population under consideration is slightly an aged population. Notably, the proportion of males aged 60 + years was higher (45%) than that of their female counterparts (29.2%).

**Table 2:** Characteristics of sampled beneficiaries that were selected as the synthetic cohort with normoglycemia for the year 2011–2012 (*N* = 1629)

	Overall		Male		Female	
Age group	**Frequency (N)**	**Percentage (%)**	**Frequency (N)**	**Percentage (%)**	**Frequency (N)**	**Percentage (%)**
30–34	50	3.1	8	1.1	42	4.6
35–39	111	6.8	32	4.4	79	8.7
40–44	198	12.2	50	6.9	148	16.3
45–49	213	13.1	85	11.8	128	14.1
50–54	250	15.3	113	15.7	137	15.1
55–59	221	13.6	111	15.4	110	12.1
60–64	209	12.8	115	16.0	94	10.3
65–69	142	8.7	66	9.2	76	8.4
70+	235	14.4	140	19.4	95	10.5
Grand Total	1629	100	720	100	909	100
Mean age (years)	55.2		58.2		52.8	
% total sampled				44.2		55.8

### Ten-year aggregate probability of getting diabetes

Table 3 shows the total number of individuals at the beginning of the year 2012 for each of the age groups, the number and proportion of individuals who turned diabetic at the end of ten years of follow-up with the probability of getting diagnosed with diabetes. As expected with an increase in age, the probability of getting diabetes increases over the period of ten years. There is around an 18% chance that an individual aged 30–34 years will progress to diabetes by the time they turn 40–44 years. The chances of an individual aged 55–59 years progressing to diabetes is as high as 59% (Table 3). The probability of getting diabetes is found to be higher among younger males aged 30–44 years as compared to females of the same age. However, the chi-square value from the log rank test indicated that there is no statistical difference in the probability of getting diabetes among males and females across age groups at any given point in time (Table 3).

**Table 3:** Overall proportion of beneficiaries diagnosed with diabetes and the probability of getting diagnosed with diabetes (with 95% CIs) over ten years of follow-up for each age group and among males and females (overall *N* = 1629)

Age group	Number of beneficiaries at baseline’	Number of beneficiaries diagnosed with Diabetes	Censored	Proportion diagnosed with diabetes in ten years	Overall Probability of getting diagnosed with diabetes (95% Cis)	Probability of getting diagnosed with diabetes (95% CIs)	
						Males	Females
30–34	50	5	45	10.0	0.18 (0.08,0.38)	0.22 (0.03,0.83)	0.17 (0.07,0.40)
35–39	111	24	87	21.6	0.36 (0.25,0.48)	0.40 (0.22,0.64)	0.34 (0.22,0.49)
40–44	198	59	139	29.8	0.46 (0.38,0.55)	0.57 (0.42,0.74)	0.42 (0.32,0.52)
45–49	213	75	138	35.2	0.52 (0.44,0.6)	0.50 (0.37,0.63)	0.54 (0.44,0.64)
50–54	250	93	157	37.2	0.54 (0.47,0.62)	0.53 (0.43,0.65)	0.55 (0.45,0.65)
55–59	221	93	128	42.1	0.59 (0.52,0.67)	0.60 (0.50,0.71)	0.58 (0.47,0.69)
60–64	209	70	139	33.5	0.50 (0.42,0.59)	0.47 (0.36,0.59)	0.54 (0.43,0.67)
65–69	142	64	78	45.1	0.62 (0.53,0.71)	0.57 (0.43,0.71)	0.67 (0.54,0.78)
70+	235	69	166	29.4	0.45 (0.38,0.54)	0.50 (0.41,0.61)	0.38 (0.27,0.51)

### Annual progression over the period of ten years

The probability of progressing to diabetes at the end of each year of follow-up for all age groups is presented in Table 4. This analysis reveals a progression pattern to diabetes over the years where 42% of the non-diabetic subjects at the onset of the study move into the diabetic state in a span of ten years. This progression pattern indicates that the shift into the diabetic state increases with age. For instance, the younger group aged 30–34 have a relatively slower progression to diabetes (16%) than those who were aged 55 at the start of the study (52%). Subjects aged between 30 and 34 years experienced their first encounter with diabetes after four years (2016) with a very low probability of 0.02, whereas older age groups developed diabetes within one year of the follow-up. Those aged 55–59 years had the highest probability of progression, i.e., a 10% chance of progression to diabetes within a year. The most revealing of this is perhaps the probability of entering the diabetic state at the end of the study period over time ages remains cumulatively greater till the age of 60 following which the systematic pattern gets violated as a major share of the older cohorts become diabetic in the middle of the study period. It was observed that if an individual has not been diagnosed with diabetes at ages 50–59 years, the probability of having diabetes at age 60–64 is almost equal to that for subjects aged 45–49 years.

**Table 4:** Probability of progressing to diabetes at the end of each of the consecutive year over ten years of follow-up among non-diabetic individuals belonging to various age-group (2012–2021)

Age group	2012	2013	2014	2015	2016	2017	2018	2019	2020	2021
30–34	≈ 0.0	≈ 0.0	≈ 0.0	≈ 0.0	0.02	0.02	0.07	0.07	0.07	0.16
35–39	≈ 0.0	0.05	0.07	0.10	0.10	0.13	0.18	0.20	0.22	0.24
40–44	≈ 0.0	0.03	0.06	0.09	0.14	0.19	0.24	0.25	0.30	0.36
45–49	≈ 0.0	0.05	0.12	0.15	0.19	0.23	0.30	0.32	0.34	0.42
50–54	≈ 0.0	0.06	0.10	0.16	0.22	0.26	0.32	0.35	0.38	0.44
55–59	≈ 0.0	0.10	0.14	0.22	0.26	0.31	0.35	0.39	0.42	0.52
60–64	≈ 0.0	0.05	0.11	0.16	0.21	0.25	0.29	0.33	0.36	0.41
65–69	≈ 0.0	0.07	0.13	0.22	0.28	0.36	0.43	0.46	0.49	0.53
70+	≈ 0.0	0.07	0.11	0.18	0.24	0.28	0.30	0.33	0.36	0.43
Overall	≈ 0.0	0.06	0.10	0.16	0.20	0.25	0.29	0.32	0.35	0.42

**Fig. 1:**
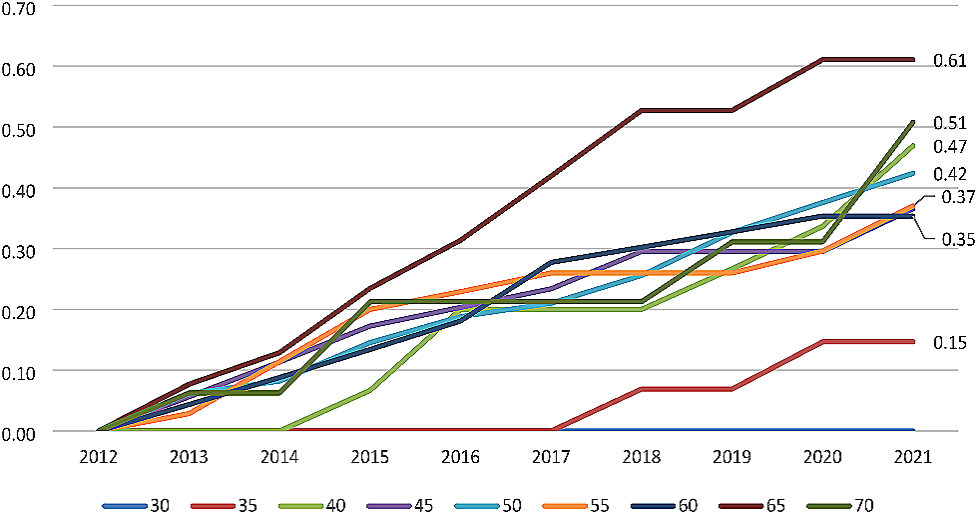
Probability of progression to diabetes over the ten years of follow-up among non-diabetic individuals at a few selected terminal ages (*N* = 270)

The annual progression pattern among males and females is presented in Table 5. Overall, 44% of males and 40% of the females progressed to diabetes in ten years. The results show that the probability of a male aged 35–39 years of developing diabetes at the end of five years (in 2016) is 16% whereas among females of the same age it is only 8%. The fastest progression over ten years was observed among males aged 55–59 years (54%) and females 65–69 years (60%). The same analysis was repeated for a few selected ages and it was found that individuals aged 30 years have nearly zero probability of developing diabetes in the next ten years, while the overall probability ranged from 15% at age 35 years to 61% at age 65 years (Fig. 1).

**Table 5:** Probability of progressing to diabetes fat the end of each of the consecutive year over ten years of follow-up among males and females

	2012	2013	2014	2015	2016	2017	2018	2019	2020	2021
**Males**										
30–34	≈ 0.0	≈ 0.0	≈ 0.0	≈ 0.0	≈ 0.0	≈ 0.0	0.15	0.15	0.15	0.15
35–39	≈ 0.0	0.09	0.13	0.16	0.16	0.22	0.22	0.25	0.25	0.25
40–44	≈ 0.0	0.06	0.12	0.16	0.25	0.29	0.34	0.38	0.38	0.46
45–49	≈ 0.0	0.02	0.07	0.12	0.20	0.24	0.28	0.29	0.30	0.42
50–54	≈ 0.0	0.06	0.11	0.16	0.22	0.27	0.32	0.36	0.37	0.42
55–59	≈ 0.0	0.06	0.10	0.16	0.23	0.28	0.32	0.37	0.41	0.54
60–64	≈ 0.0	0.04	0.08	0.15	0.21	0.22	0.24	0.30	0.32	0.37
65–69	≈ 0.0	0.05	0.10	0.16	0.24	0.33	0.36	0.40	0.42	0.45
70+	≈ 0.0	0.06	0.11	0.18	0.24	0.29	0.31	0.37	0.42	0.49
Overall	≈ 0.0	0.05	0.10	0.16	0.22	0.27	0.30	0.34	0.36	0.44
**Females**										
Age group	2012	2013	2014	2015	2016	2017	2018	2019	2020	2021
30–34	≈ 0.0	≈ 0.0	≈ 0.0	≈ 0.0	0.03	0.03	0.05	0.05	0.05	0.16
35–39	≈ 0.0	0.03	0.05	0.08	0.08	0.09	0.16	0.17	0.20	0.23
40–44	≈ 0.0	0.02	0.04	0.06	0.10	0.16	0.20	0.21	0.27	0.32
45–49	≈ 0.0	0.06	0.15	0.17	0.18	0.22	0.31	0.33	0.36	0.42
50–54	≈ 0.0	0.06	0.09	0.17	0.21	0.26	0.32	0.34	0.38	0.47
55–59	≈ 0.0	0.14	0.18	0.29	0.29	0.34	0.38	0.41	0.42	0.50
60–64	≈ 0.0	0.14	0.18	0.29	0.29	0.34	0.38	0.41	0.42	0.50
65–69	≈ 0.0	0.10	0.16	0.27	0.32	0.38	0.49	0.51	0.57	0.60
70+	≈ 0.0	0.07	0.11	0.19	0.25	0.26	0.28	0.28	0.28	0.32
Overall	≈ 0.0	0.06	0.11	0.16	0.19	0.23	0.29	0.31	0.34	0.40

### A life table for a synthetic cohort of the non-diabetic population

Using the ten-year probability of progressing to diabetes (from Table 3), an abridged life table was constructed to estimate the expected number of years remaining without being diagnosed with diabetes for individuals free from diabetes before that age. It was found that an individual aged 30–34 years has 11.9 years without being diagnosed with diabetes, implying that the probability of having diabetes is almost negligible till he/she turns 40–44 years. The estimated age at onset is therefore around 42 years in this urban community. An individual aged 50–54 years is expected to have around 6.3 years before progressing to diabetes, whereas an individual aged 65–69 years is only expected to live for 3.5 years more without being diagnosed with diabetes (Table 6).

**Table 6:** Expected number of years lived before progressing to diabetes by non-diabetic individuals at various ages

Age group as of year 2012	Ten-year Probability of of remaining free from diabetes	Probability of getting diagnosed with Diabetes in next 10 years	Number of beneficiaries surviving at the start of year	Person years		Expected number of remaining years without Diabetes
	px	qx	lx	Lx	Tx	ex
30–34	0.82	0.18	1000	4545.5	11854.3	11.9
35–39	0.64	0.36	818	3363.6	7308.8	8.9
40–44	0.54	0.46	527	2031.1	3945.2	7.5
45–49	0.48	0.52	285	1054.6	1914.1	6.7
50–54	0.46	0.54	137	498.0	859.5	6.3
55–59	0.41	0.59	63	220.1	361.5	5.8
60–64	0.50	0.50	25	95.5	141.4	5.5
65–69	0.38	0.62	13	43.8	45.9	3.6
70+	0.55	0.45	5	2.1	2.1	0.4

*Note* nqx: probability of progressing to diabetes between age x to x + n

lx: number of individuals surviving without diabetes at the beginning of age x

nLx: person years lived without diabetes by lx individuals in the age group x to x + n

Tx: person years lived without diabetes by the individuals after exact age x

ex: Average number of expected years a person aged x is expected to live free of diabetes/before progressing to diabetes

Females aged 30–34 years have around 12.3 years more without diabetes diagnosis whereas males of the same age are expected to live around 10.6 more years before progressing to diabetes. These results reveal that males have a shorter waiting time and faster progression to diabetes in comparison to females. The number of years before progressing to diabetes is higher among females up to the age of 45 years only, and later, the pattern changes though the difference in the number of years before being diagnosed with diabetes among males and females isn’t statistically significant (Table 7).

**Table 7:** Expected number of years lived before progressing to diabetes by non-diabetic males and females at various ages

	Males					Female				
Age group	qx	lx	Lx	Tx	ex	qx	lx	Lx	Tx	ex
30–34	0.22	1000	4444.5	10618.9	10.6	0.17	1000	4565.3	12253.5	12.3
35–39	0.40	778	3111.2	6174.4	7.9	0.34	826	3434.9	7688.2	9.3
40–44	0.57	467	1666.7	3063.2	6.6	0.42	548	2168.1	4253.3	7.8
45–49	0.50	200	752.3	1396.5	7.0	0.54	319	1168.0	2085.3	6.5
50–54	0.53	101	370.1	644.2	6.4	0.55	148	535.8	917.3	6.2
55–59	0.60	47	164.6	274.1	5.8	0.58	66	235.9	381.5	5.7
60–64	0.47	19	71.6	109.4	5.9	0.54	28	101.6	145.6	5.2
65–69	0.57	10	35.7	37.8	3.8	0.67	13	42.5	44.0	3.5
70+	0.50	4	2.1	2.1	0.5	0.38	4	1.5	1.5	0.4

*Note* nqx: probability of progressing to diabetes between age x to x + n

lx: number of individuals surviving without diabetes at the beginning of age x

nLx: person years lived without diabetes by lx individuals in the age group x to x + n

Tx: person years lived without diabetes by the individuals after exact age x

ex: Average number of years a person aged x is expected to live free of diabetes/before progressing to diabetes

## Discussion

Diabetes is a chronic disease that significantly increases the risk of developing other potentially life-threatening conditions. Due to shifts in urbanization and lifestyle factors, there’s been a noted change in the onset of diabetes, with the condition now affecting a younger demographic [22, 32]. In light of the limited available evidence regarding the progression and risk of diabetes onset, this study aimed to estimate the likelihood of transitioning to diabetes within a ten-year period among a non-diabetic synthetic cohort. Additionally, the research sought to examine variations in this progression among different age groups and between males and females, employing a life table approach for analysis. The study revealed that, over a ten-year period, 42% of the beneficiaries in the study population were diagnosed with diabetes. Females had a lower likelihood of developing diabetes compared to males. In a recent publication authored by Sharma and colleagues, an investigation within the same study population revealed a lifetime diabetes risk estimated at 40% [19]. This observation underscores the considerable susceptibility of the community to diabetes, indicating a high rate of diabetes incidence within this demographic. Additionally, a parallel study on the lifetime risk of diabetes conducted by Luhar et al. demonstrated a decline in the remaining lifetime risk (95% confidence interval) with age, reaching 37.7% at the age of 60 for women and 27.5% for men [33]. In a previous investigation involving Asian Indians, it was discovered that over a ten-year period, the incidence of individuals progressing from impaired glucose tolerance (IGT) to diabetes varied between 13% and 52% [9]. Another study conducted in India, employing the life table methodology, revealed that the likelihood of maintaining a diabetes-free life diminishes with age. Specifically, only 30% of the overall participants remained free of diabetes beyond the age of 77 [23].

The current study observed that males had a slightly higher likelihood of developing diabetes compared to females. However, it’s important to note that these differences in diabetes risk between males and females were not found statistically significant across various age groups at any point during the follow-up period of ten years. Consistent with our findings, several previous studies have also noted an elevated diabetes risk among males [10–18]. An Indian study specifically highlighted that the likelihood of remaining diabetes-free is notably lower for men compared to women, particularly for urban residents and those belonging to higher socioeconomic classes [23]. This investigation utilized data from the India Human Development Survey (IHDS) and discovered that only 26% of urban males and 33% of urban females remained diabetes-free until the age of 73 in India [23]. It has been argued that the observed discrepancy in survival probabilities can possibly be attributed to the high male mortality in the non-diabetic population [34]. It has also been found that the western parts of India (including Maharashtra and Goa), in comparison to other regions of India, had an overall higher probability of diabetes progression, which was around 71% for males and 79% for females [23].

As age increased, the likelihood of developing diabetes also rose, peaking at around 59 years of age. This age-related pattern aligns with findings from prior research studies. Notably, the highest probability of diabetes progression was observed among older men (55–59 years) and women (65–69 years), consistent with earlier investigations [10, 13]. While some studies have reported variations in the precise age peak for diabetes incidence, most have identified the highest incidence of type II diabetes in men aged 55–64 years and women aged 65–69 years, with some indicating an overall peak occurring at 55–59 years [10, 13, 15]. For instance, a study led by Singh and colleagues identified the highest likelihood of developing diabetes between the ages of 38 and 58, with an 8% probability [23]. Another ten-year study found that the risk of diabetes increased until the age of 75, after which it declined. Importantly, this study underscored that the rate of progression from pre-diabetes to diabetes among older adults was one-third of that seen in middle-aged individuals. This suggests that, at later stages of life, the probability of transitioning to diabetes is lower when compared to the higher risk observed in middle-aged individuals [35].

Diabetes is typically a condition that doesn’t show noticeable symptoms, and tends to develop slowly, sometimes taking several years to manifest, making it easy to overlook. Consequently, this study utilized a ten-year age and sex stratified approach to estimate the probability of progressing to diabetes. Also, it then constructed a synthetic cohort to estimate the additional years a person can expect to live before a diabetes diagnosis. The study found that the population aged 30–34 years is expected to live around 11.9 years before progressing to diabetes, yielding the age at onset to be around 42 years. The onset of diabetes in our study was observed to occur at an age that is relatively early compared to some other Indian studies. For instance, a ten-year prospective study conducted in Kerala, India, found that the average age at which individuals developed diabetes was 47.3 years [12]. The Chennai Urban-Rural Epidemiological study (CURES study) reported the mean age at diagnosis of diabetes among the incident diabetes cases to be 50.9 ± 12.8 years [26]. Studies have also reported that the onset of diabetes in urban India is about a decade earlier than among their western counterparts [36]. The study found a faster progression to diabetes among males, which is in line with previous studies. It has been reported that males are at a higher risk of developing diabetes at an earlier age than females, i.e. age at onset of newly diagnosed diabetes is earlier among males [13, 15].

The observed variations in the estimates can be predominantly attributed to disparities in the age and sex demographics of the current study population compared to those in previous studies as a majority of the mentioned studies have focused on populations aged 20 years and above. Also, research has demonstrated that the principal factors associated with the progression to diabetes in India are urbanization and elevated economic status [23], a trend that is also apparent in the distribution pattern of diabetes-free survival within our study. Furthermore, these elevated estimates in our study may also be attributed to distinctive factors, including an urban-dwelling population, moderate to high levels of educational attainment, economic stability, and the presence of universal and uniform access to healthcare. Moreover, the study’s population benefits from a preventative and universal access medical scheme with rigorous screening and diagnostic evaluations. This unique characteristic partly explains the faster diabetes progression observed in this urban community.

Disease progression models often rely on long-term patient observations, but in India, such longitudinal studies are limited due to extended follow-up periods and high associated costs [37–39]. This study stands out in that it utilizes data from an Electronic Medical Record system within a hospital, leveraging clinically confirmed incident diabetes cases to estimate diabetes progression over a decade of follow-up. The study’s findings have significant implications for early diabetes detection and the optimal age to commence diabetes screening in the population. While the American Diabetes Association (ADA) currently recommends regular screening for individuals aged 45 and above, evidence from this urban community suggests a need to reassess the starting age for diabetes screening in India. The study corroborates the need to rigorously implement the national guideline to screen all adults aged 30 years and above for diabetes. The screening capacity of health care systems needs to be strengthened exponentially to reach out to these age-groups in the populations [40]. This approach can aid in identifying high-risk or pre-diabetic individuals and may contribute to delaying diabetes onset through prescribed interventions. These data and its outcomes hold substantial importance for implementing preventive strategies against diabetes among a large patient population, with far-reaching economic, social, and psychological implications. Furthermore, such real-time data-driven decisions can serve as compelling case studies in the Indian context, illustrating potential strategies for early detection and diabetes prevention based on estimated onset age and observed progression patterns.

### Limitations of the study

The study has a few limitations that warrant acknowledgment. Firstly, it’s important to recognize that the study population consisted of individuals who were beneficiaries of the Contributory Health Service Scheme and received healthcare services under a uniform system. Hence, it’s essential to exercise caution when attempting to generalize the findings of this study to other regions of the country, as the incidence and progression of diabetes can differ across different settings. Secondly, when comparing our results with those of previous studies, caution is advised due to variations in the age and sex distributions of the populations under study, as well as differences in the definitions used for diabetes. Lastly, the study was unable to account for other factors that can influence the development and progression of diabetes, such as diet, obesity, physical inactivity, and the presence of various comorbid conditions. Consequently, establishing a causal relationship between these variables and diabetes was not feasible within the scope of this study.

## Data Availability

The datasets generated and/or analysed during the current study are not publicly available due to data privacy norms of the hospital under consideration. Data are however available from the corresponding author upon reasonable request to pal3193@gmail.com and with permission of Medical Division, Bhabha Atomic Research Center, Mumbai, after due approvals.

## Abbreviations

ADA: American Diabetes Association
BMI: Body mass index
CHSS: Contributory Health Service Scheme
CURES: Chennai urban-rural epidemiological study
EMR: Electronic medical records
FPG: Fasting plasma glucose
HbA1c: Glycated hemoglobin
IFG: Impaired fasting glucose
IGT: Impaired glucose tolerance
IHDS: India Human Development survey
NCDs: Non-communicable diseases
PPG: Postprandial glucose
